# Supplementary material for: Health Economic Evaluation of an Online-Based Motivational Program to Reduce Problematic Media Use and Promote Treatment Motivation for Internet Use Disorder—Results of the OMPRIS Study
Source: Int J Environ Res Public Health. 2023 Dec 5;20(24):7144. doi: 10.3390/ijerph20247144 (PMC10742498; doi:10.3390/ijerph20247144)
Supplement: Supplementary file 1 [file ijerph-20-07144-s001.zip › ijerph-2640428-supplementary.pdf]

International Journal of
Environmental Research and Public Health

MDPI

Supplementary Material

Table S1. Additional baseline characteristics.

Sociodemographic variables	total (n = 169)	IG (n = 81)	WCG (n = 88)
**Living situation [n(%)]**			
alone	41 (24.3)	19 (23.5)	22 (25.0)
with partner	48 (28.4)	23 (28.4)	25 (28.4)
in a flat-sharing community	46 (27.2)	23 (28.4)	23 (26.1)
with a family member	32 (18.9)	14 (17.3)	18 (20.5)
in a supervised flat-sharing community	2 (1.2)	2 (2.5)	0
**Marital status [n (%)]**			
unmarried without partner	88 (52.1)	41 (50.6)	47 (53.4)
unmarried with partner	39 (23.1)	20 (24.7)	19 (21.6)
married/cohabitation	35 (20.7)	16 (19.8)	19 (21.6)
divorced/separated	5 (3.0)	3 (3.7)	2 (2.3)
widowed	2 (1.2)	1 (1.2)	1 (1.1)
**Vocational qualification [n (%)]**			
still in training/apprenticeship	5 (3.0)	2 (2.5)	3 (3.4)
still studying at the university	67 (39.6)	31 (38.3)	36 (40.9)
completed apprenticeship	26 (15.4)	10 (12.3)	16 (18.2)
completed studying at the university	60 (35.5)	32 (39.5)	28 (31.8)
none	9 (5.3)	5 (6.2)	4 (4.5)
other	2 (1.2)	1 (1.2)	1 (1.1)
**Health insurance [n (%)]**			
statutory	142 (84.0)	70 (86.4)	72 (81.8)
private (full insurance)	19 (11.2)	8 (9.9)	11 (12.5)
government allowance for public employees/ private	6 (3.6)	3 (3.7)	3 (3.4)
none	2 (1.2)	0	2 (2.3)
**Exemption from additional payments [n (%)]**			
no	151 (89.3)	72 (88.9)	79 (89.8)
yes	18 (10.7)	9 (11.1)	9 (10.2)

IG = Intervention Group; WCG = Waiting Control Group.

Table S2. Utilization of medical care at T0 and T2 for the total group, the IG, and the WCG from the perspective of the statutory health insurance.

Resource categories	T0				T2			
	total n (% PU)	IG n (% PU)	WCG n (% PU)	p-value	total n (% PU)	IG n (% PU)	WCG n (% PU)	p-value
Sickness benefits	2 (1.18)	0	2 (2.27)	0.498	2 (1.18)	0	2 (2.27)	0.498
Outpatient physician contacts	111 (65.68)	54 (66.67)	57 (64.77)	0.872	78 (46.15)	37 (45.68)	41 (46.59)	1.000
Hospital treatments	6 (3.55)	4 (4.94)	2 (2.27)	0.428	3 (1.78)	1 (1.23)	2 (2.27)	1.000
Medications	59 (34.91)	25 (30.86)	34 (38.64)	0.334	50 (29.59)	23 (28.40)	27 (30.68)	0.866
Remedies	17 (10.06)	8 (9.88)	9 (10.23)	1.000	7 (4.14)	4 (4.94)	3 (3.41)	0.711

PU = persons with utilization.

Table S3. Utilization of incapacity to work, reduction in earning capacity and medication at T0 and T2 for the total group, the IG, and the WCG from the societal perspective).

Resource categories	T0				T2			
	total n (% PU)	IG n (% PU)	WCG n (% PU)	p-value	total n (% PU)	IG n (% PU)	WCG n (% PU)	p-value
Incapacity to work	7 (4.14)	2 (2.47)	5 (5.68)	0.446	8 (4.73)	2 (2.47)	6 (6.82)	0.281
Reduction in earning capacity	2 (1.18)	0	2 (2.27)	0.498	3 (1.78)	1 (1.23)	2 (2.27)	1.000
Medications	64 (37.9)	26 (32.1)	38 (43.18)	0,155	57 (33.73)	26 (32.1)	31 (35.23)	0.745

Table S4. Financial burdens of participants and use of resources from the public sectors' point of view of the total group, the IG, and the WCG at T0.

Use of resources at T0 [n (%)]	total (n = 169) [n (% PU)]	IG (n = 81) [n (% PU)]	WCG (n = 88) [n (% PU)]	p-value
**Use of cash benefits**				
job center	5 (2.96)	2 (2.47)	3 (3.41)	1.000
employment agency	3 (1.78)	3 (3.70)	0 (0)	0.108
Federal Education and Trainings Assistance Act (“BAföG”)	9 (5.32)	3 (3.70)	6 (10.23)	0.499
scholarships	4 (2.37)	2 (2.47)	2 (2.27)	1.000
support from parents	58 (34.32)	28 (34.57)	30 (34.09)	1.000
other	25 (14.79)	8 (9.88)	17 (19.32)	0.361
**Debts due to**				
online gaming	1 (0.59)	1 (1.23)	0 (0)	0.479
internet utilization	0 (0)	0 (0)	0 (0)	-
rent debts	2 (1.18)	0 (0)	2 (2.27)	0.498
unpaid bills	0 (0)	0 (0)	0 (0)	-
consumer credit	11 (6.51)	6 (7.41)	5 (5.68)	0.759
overdraft facility	5 (2.96)	3 (3.70)	2 (2.27)	0.671
other	26 (15.38)	10 (12.35)	16 (18.18)	0.394
**Vocational rehabilitation measures**				
vocational preparation programs	0 (0)	0 (0)	0 (0)	-
vocational programs in fields outside the company	0 (0)	0 (0)	0 (0)	-
job coaching	0 (0)	0 (0)	0 (0)	-
rehabilitation for mentally ill people	3 (1.78)	2 (2.47)	1 (1.14)	0.608
programs for reintegration into working	1 (0.59)	1 (1.23)	0 (0)	0.479
protected factory: work training area	0 (0)	0 (0)	0 (0)	-
protected factory: work area	0 (0)	0 (0)	0 (0)	-
integration workplace	0 (0)	0 (0)	0 (0)	-
supported employment: individual in-company qualification	0 (0)	0 (0)	0 (0)	-
professional training	0 (0)	0 (0)	0 (0)	-
supported employment: vocational support	0 (0)	0 (0)	0 (0)	-
integration specialist service/psychosocial specialist service	0 (0)	0 (0)	0 (0)	-
suitability assessment/career guidance	0 (0)	0 (0)	0 (0)	-
other	0 (0)	0 (0)	0 (0)	-
**Utilization of special offers**				
counseling center for mental health problems/addiction counseling center	19 (11.24)	10 (12.35)	9 (10.23)	0.808
social psychiatric service / service for psychological and social crises	1 (0.59)	0 (0)	1 (1.14)	1.000
day care centers/contact centers for people with mental health problems	1 (0.59)	1 (1.23)	0 (0)	0.479
self-help groups because of mental suffering	10 (5.92)	5 (6.17)	5 (5.68)	1.000
offers because of psychological issues (e.g. helpline)	5 (5.68)	2 (2.47)	3 (3.41)	1.000

Table S5. Financial burdens of participants and use of resources from the public sectors' point of view of the total group, the IG, and the WCG at T2.

Use of resources at T2 [n (%)]	total (n = 169) n (% PU)	IG (n = 81) n (% PU)	WCG (n = 88) n (% PU)	p-value
**Use of cash benefits**				
job center	4 (2.37)	2 (2.47)	2 (2.27)	1.000
employment agency	2 (1.18)	2 (2.47)	0 (0)	0.228
Federal Education and Trainings Assistance Act ("BAföG")	10 (5.92)	3 (3.70)	7 (7.95)	0.333
scholarships	5 (2.96)	3 (3.70)	2 (2.27)	0.671
support from parents	49 (29.59)	24 (30.86)	25 (28.41)	0.867
other	16 (9.47)	8 (9.88)	8 (9.09)	1.000
**Debts due to**				
online gaming	1 (0.59)	1 (1.23)	0 (0)	0.479
internet utilization	0 (0)	0 (0)	0 (0)	-
rent debts	1 (0.59)	0 (0)	1 (1.14)	1.000
unpaid bills	1 (0.59)	1 (1.23)	0 (0)	0.479
consumer credit	10 (5.92)	6 (7.40)	4 (4.55)	0.523
overdraft facility	5 (2.96)	3 (3.70)	2 (2.27)	0.671
other	22 (13.02)	8 (9.88)	14 (15.90)	
**Vocational rehabilitation measures**				
vocational preparation programs	0 (0)	0 (0)	0 (0)	-
vocational programs in fields outside the company	0 (0)	0 (0)	0 (0)	-
job coaching	0 (0)	0 (0)	0 (0)	-
rehabilitation for mentally ill people	1 (0.59)	1 (1.23)	0 (0)	0.479
programs for reintegration into working	0 (0)	0 (0)	0 (0)	
protected factory: work training area	0 (0)	0 (0)	0 (0)	-
protected factory: work area	0 (0)	0 (0)	0 (0)	-
integration workplace	0 (0)	0 (0)	0 (0)	-
supported employment: individual in-company qualification	0 (0)	0 (0)	0 (0)	-
professional training	0 (0)	0 (0)	0 (0)	-
supported employment: vocational support	0 (0)	0 (0)	0 (0)	-
integration specialist service/psychosocial specialist service	0 (0)	0 (0)	0 (0)	-
suitability assessment/career guidance	0 (0)	0 (0)	0 (0)	-
other	0 (0)	0 (0)	0 (0)	-
**Utilization of special offers**				
counseling center for mental health problems/addiction counseling center	14 (8.28)	10 (1.23)	4 (4.54)	0.093
social psychiatric service / service for psychological and social crises	0 (0)	0 (0)	0 (0)	-
day care centers/contact centers for people with mental health problems	1 (0.59)	1 (1.23)	0 (0)	0.479
self-help groups because of mental suffering	5 (2.96)	3 (3.70)	2 (2.27)	0.671
offers because of psychological issues (e.g. helpline)	1 (0.59)	1 (1.23)	0 (0)	0.479

Table S6. Pricing of resource use including references.

Resource	Unit	SHI perspective	Societal perspective	Reference
**Incapacity to work**	EUR/day	-	189.94[a, b]	[28]
**Sickness benefit according to §47 (SGB V)**	EUR/day	126.77[a]	-	[17,28]
**Reduction in earning capacity**	EUR/day	-	189.94[a, b]	[28]
**General practitioner**	EUR/contact	21.81[c]	22.81	[9]
**Ophthalmologist**	EUR/contact	37.81[c]	39.54	
**Surgeon**	EUR/contact	47.17[c]	49.33	
**Gynecologist**	EUR/contact	32.75[c]	34.26	
**Dermatologist**	EUR/contact	20.54[c]	21.48	
**Otolaryngologist**	EUR/contact	28.70[c]	30.02	
**Psychiatric outpatient clinic**	EUR/contact	74.54[c]	77.82	[35,36]
**Internist**	EUR/contact	71.14[c]	74.41	[9]
**Pediatrician**	EUR/contact	37.33[c]	39.04	
**Neurologist**	EUR/contact	48.61[c]	50.85	
**Orthopedist**	EUR/contact	27.63[c]	28.90	
**Psychiatrist**	EUR/contact	48.61[c]	50.85	
**Psychotherapist**	EUR/contact	84.88[c]	88.78	
**Urologist**	EUR/contact	26.85[c]	28.08	
**Dentist**	EUR/contact	60.74[c]	63.52	
**Other specialties [d]**	EUR/contact	42.47[c]	44.42	
**Hospital inpatient: psychiatric-psychotherapeutic station / psychosomatic-psychotherapeutic station**	EUR/day	305.67[e]	386.25	
**Hospital inpatient: other sections (primarily physical illnesses/treatments)**	EUR/day	574.22[e]	654.80	
**Hospital semi-inpatient (day/night-hospital): psychiatric-psychotherapeutic station / psychosomatic-psychotherapeutic station**	EUR/day	170.48[e]	251.06	
**Hospital semi-inpatient: other sections (primarily physical illnesses/treatments)**	EUR/day	345.03[e]	425.61	
**Rehabilitation inpatient: addictive disease/ weaning treatment**	EUR/day	-	135.46	[10]
**Rehabilitation inpatient: other mental illness**	EUR/day	-	122.53	
**Rehabilitation inpatient: other sections (primarily physical illnesses/treatments)**	EUR/day	-	138.54	[9]
**Rehabilitation semi-inpatient (day/night-hospital): Addictive disease/ weaning treatment**	EUR/rehabilitation	-	4233.25	[10]
**Rehabilitation semi-inpatient (day/night-hospital): other mental illness**	EUR/rehabilitation	-	4612.07	
**Rehabilitation semi-inpatient (day/night-hospital): other sections (primarily physical illnesses/treatments)**	EUR/day	-	53.08	[9]
**Physiotherapy**	EUR/contact	17.57[f]	18.67	
**Logopedics**	EUR/contact	41.29[f]	43.88	
**Occupational therapy**	EUR/contact	40.14[f]	42.65	
**Sociotherapy**	EUR/contact	18.53[f]	19.69	[10]
**Psychoeducation**	EUR/contact	-	45.86	
**Dance therapy/movement therapy - Individual**	EUR/contact	-	63.75	
**Dance therapy/movement therapy - Group**	EUR/contact	-	22.02	
**Art therapy - Individual**	EUR/contact	-	74.76	
**Art therapy - Group**	EUR/contact	-	43.28	
**Music therapy - Individual**	EUR/contact	-	61.86	
**Music therapy - Group**	EUR/contact	-	27.98	

**Theatrical therapy - Individual**	EUR/contact	-	64.90	
**Theatrical therapy - Group**	EUR/contact	-	23.90	
**Relaxation techniques - Individual**	EUR/contact	36.31	38.58[g]	[13]
**Relaxation techniques - Group**	EUR/contact	9.75	10.37[g]	
**Relatives or friends (due to mental illness)**	EUR/hour	-	23.98	[9]
**Paid domestic help (due to mental illness)**	EUR/hour	-	15.07	[29]
**Child care (due to mental illness)**	EUR/hour	-	19.57	[30]
**Legal representative**	EUR/hour	-	29.33	[31]
**Social worker or accompanying person**	EUR/hour	-	29.33	[32]
**Youth Welfare Office**	EUR/hour	-	24.09	[33]
**Outpatient psychiatric nursing service**	EUR/hour	-	61.07	[10]

a Including employer's contribution factor 1.28 [17]; b Friction cost approach (average vacancy period 2021 = 122 days) [34]; c Conversion formula (eliminating part of private health insurance) societal to SHI perspective: SHI [EUR] = society [EUR] * (1-0.0439) [9]; d Average outpatient physician contacts excluding psychiatric outpatient clinic; e Conversion formula (subtracting investment costs so that only operating costs are included) societal to SHI perspective: SHI [EUR] = society [EUR] – EUR 70.87 [9]; f Conversion formula (eliminating percentage of private health insurance) societal to SHI perspective: SHI = society [€]/1,0626 [9]; g Conversion formula (adding percentage of private health insurance) SHI to societal perspective: Societal = SHI*1.0626 [9].

Legend:

**SHI**	Statutory Health Insurance
**SGB V**	(German) Social Security Code V
